# Low Birth Weight Disturbs the Intestinal Redox Status and Mitochondrial Morphology and Functions in Newborn Piglets

**DOI:** 10.3390/ani11092561

**Published:** 2021-08-31

**Authors:** Jiaojiao Chen, Yi Song, Daiwen Chen, Bing Yu, Jun He, Xiangbing Mao, Zhiqing Huang, Junqiu Luo, Jie Yu, Yuheng Luo, Hui Yan, Ping Zheng

**Affiliations:** Key Laboratory for Animal Disease-Resistance Nutrition of China Ministry of Education, Institute of Animal Nutrition, Sichuan Agricultural University, Chengdu 611130, China; c17685341905@163.com (J.C.); 15283507010@163.com (Y.S.); chendwz@sicau.edu.cn (D.C.); ybingtian@163.com (B.Y.); hejun8067@163.com (J.H.); acatmxb2003@163.com (X.M.); zqhuang@sicau.edu.cn (Z.H.); junqluo2018@tom.com (J.L.); jerryyujie@163.com (J.Y.); luoluo212@126.com (Y.L.); yan.hui@sicau.edu.cn (H.Y.)

**Keywords:** jejunum, low birth weight, mitochondria, piglets, redox status

## Abstract

**Simple Summary:**

Low birth-weight piglets normally have a higher growth retardation and are more prone to disease such as diarrhea compared to NBW piglets, which are strongly associated with intestinal health, body redox status and mitochondrial morphology and function. The present study showed that low birth-weight piglets exhibited abnormal intestinal development and impaired intestinal barrier function and redox status when compared to normal- birth-weight piglets. Furthermore, we found that the impaired mitochondrial structure and functions may be one of the main causes of intestinal dysfunction in low birth-weight piglets. These results provided insights for the mechanisms of intestinal dysfunction in low birth-weight piglets.

**Abstract:**

Low birth-weight (LBW) neonates exhibit a lower growth rate and impaired intestinal development. However, the reasons for abnormal development of small intestine in LBW piglets have not been widely studied. The present study focused on the redox status and mitochondrial morphology and functions of the small intestine in LBW newborn piglets. Ten newborn normal birth-weight (NBW) piglets and LBW piglets from 10 primiparous sows with the same parturition day were selected and sampled immediately without sucking colostrum. The small intestine tissues were collected and measured. Compared with NBW newborn piglets, LBW newborn piglets had a significantly decreased length and weight of the small intestine (*p* < 0.05) as well as the villus height/crypt depth (V/C) index in the jejunum (*p* < 0.05). Furthermore, LBW piglets had a lower gene expression of tight junction protein zonula occluden-1 (*ZO1*), *claudin 1*, antioxidant enzyme catalase (*CAT*), glutathione peroxidase (*GPX*) and heme oxygenase-1 (*HO-1*) in jejunum (*p* < 0.05). Meanwhile, LBW induced mitochondrial vacuolation and significantly decreased the mRNA expression of PPARγ coactivator-1α (*PGC-1α*) (*p* < 0.05) and tended to decrease the expression of cytochrome coxidase IV (*Ccox IV*) (*p* = 0.07) and cytochrome C (*Cytc*) (*p* = 0.08). In conclusion, LBW newborn piglets showed an abnormal development of the small intestine and disturbed redox status, and this may be caused by impaired morphology and the functions of mitochondria in the jejunum.

## 1. Introduction

In the past decade, the average litter size of pigs had been increased gradually by genetic selection associated with the reduction of the mean piglets’ birth weight and the rise in the proportion of small piglets [1]. The percentage of low-birth-weight (LBW) piglets (less than 1.0 kg at birth) increased from 7% to 23% in the case of 11 and 15 piglets, respectively, per litter [2,3]. Studies indicated that impaired uterine capacity, blood flow, and nutrient availability resulted in impaired growth and development of the embryo and fetus and led to LBW piglets [1,4]. LBW commonly occurs in humans and mammals and is associated with an increased risk of newborn morbidity and mortality as well as impaired postnatal growth performance [5,6].

The small intestine is critical in nutrient metabolism and as a defensive barrier. Previous studies reported that the poor growth of LBW piglets was partly due to the impairment of digestive and absorptive functions in the small intestine [7,8]. Then studies indicated that the dysfunction of small intestine of LBW piglets is associated with a longer and thinner intestine [9], lower villi height, thinner mucosa and muscle layers [10], as well as higher intestinal permeability in the small intestine at birth [11]. Furthermore, recent research demonstrated that altered proteomes with increased abundances of protein associated with oxidative stress and apoptosis, as well as decreased protein associated with cell structure and absorption of nutrients, might be the major reason responsible for the dysfunctional intestine in LBW piglets [12]. The neonates are susceptible to oxidative stress because of the high level of free radicals and underdevelopment of the antioxidant system in the intestine at birth [13,14]. In addition, LBW piglets had lower expression of antioxidant enzymes such as heme oxygenase, catalase and thioredoxin in the small intestine compared to NBW piglets at birth [11]. Taken together, LBW piglets had a dysfunction of the intestine with underdeveloped morphology, higher permeability, free radicals and lower antioxidant capacity. However, the underlying mechanisms of intestinal dysfunction in LBW piglets remain unclear.

Mitochondria play a central role in energy metabolism of the body and mitochondrial function is important in maintaining the cellular integrity. At the same time, mitochondria are one of the important sites of endogenous reactive oxygen species (ROS) generation [15,16]. When the content of ROS in the body exceeds its clear ability, it will lead to oxidative stress. However, few studies focus on the oxidative stress and mitochondria biogenesis in the small intestine of LBW neonates. LBW newborn piglets showed an abnormal development of small intestine and disturbed redox status, and this may be caused by impaired morphology and function of mitochondria in the intestine. Therefore, the aim of this study was to investigate the effects of LBW on the intestinal development and the mitochondria biogenesis and structure in the jejunum of newborn piglets.

## 2. Materials and Methods

### 2.1. Animals and Experimental Design

The body weight of all piglets was recorded at the time of birth. NBW and LBW piglets could be defined following the criteria according to the description of Michiels et al. [17]. Ten pairs of male NBW newborn piglets with average birth weight of 1.66 ± 0.07 kg (1.60–2.0 kg) and LBW newborn piglets with average birth weight of 0.83 ± 0.04 kg (0.60–1.0 kg) were obtained at birth without eating colostrum from 10 sows (Large White × Landrance) in a pig farm (Mianyang, China).

### 2.2. Sample Collection

Blood samples were collected from jugular vein into glass tubes without anticoagulant, centrifuged at 3000 g/min for 15 min at 4 °C, serum (supernatant) samples were collected and stored at −20 °C for further analyses.

After the blood was collected, piglets were euthanized with intravenous injection of pentobarbital sodium (50 mg/kg BW) and then slaughtered by exsanguination protocols approved by the Sichuan Agricultural University Animal Care Advisory Committee. The abdomen was incised and the entire small intestine from pylorus to ileocecal valve was removed. The length and weight of the intestine were measured and recorded. The small intestine was divided into three segments: duodenum, jejunum and ileum as we previously described [18]. Nearly 2 cm samples in the middle part of the duodenum, jejunum and ileum were obtained and flushed gently with ice-cold PBS (pH 7.4) and then stored in 4% paraformaldehyde solution (Adamas, Shanghai, China) for histological analysis. Approximately 0.5 mm^3^ jejunal segments were collected and stored on ice immediately and washed with PBS (pH 7.4), then fixed in 2.5% buffered glutaraldehyde (Yuanye, Shanghai, China) for mitochondria transmission electron microscope analysis. Following this, 2 cm jejunum samples were snap frozen in liquid nitrogen and stored at −80 °C for PCR analysis.

### 2.3. Determination of Histological Analyses

Duodenum, jejunum and ileum samples were fixated in 4% paraformaldehyde solution, embedded by paraffin and stained with haematoxylin and eosin. A total of 30 well-oriented villi of three visual fields were picked per piglet. Villus heights and crypt depths were measured using an image processing and analysis system (Optimus software version 6.5, Media Cybergenetics, Bethesda, MD, USA). The corresponding villus height/crypt depth (V/C) index was calculated.

Jejunal tissue was cut into approximately 1 mm^3^ pieces, fixed in 2.5% glutaraldehyde, rinsed in 0.1 mol/L sodium phosphate buffer (pH 7.4) three times for 15 min each, and then osmicated in 1% osmic acid for 3 h at 4 °C. After dehydration with a graded ethanol series, the sample was embedded and sectioned into 70 nm slices. Sections were viewed and photographed in transmission electron microscopes using a JEM-1230 (JEOL Co. Ltd., Tokyo, Japan).

### 2.4. Enzyme Activities Analysis

Jejunal mitochondria were isolated using the gradient centrifugation method by assay kit from Nanjing Jiancheng Bioengineering Institute (Nanjing, Jiangsu, China). The activities of catalase (CAT) and superoxide dismutase (SOD) and the concentrations of malondialdehyde (MDA) in jejunal mitochondria were determined using assay kits from Nanjing Jiancheng Bioengineering Institute (Nanjing, Jiangsu, China). The methods were according to the manufacturer’s instructions. The results were expressed as “U/mg protein”.

### 2.5. Determination of Gene mRNA Expression

Total RNA of the jejunum sample was extracted using Trizol reagent (TaKaRa, Dalian, China) according to the manufacturer’s instructions. The concentration (ranging between 600 to 1300 ng/µL) and purity (A260/280 ranging between 1.9 to 2.1) of RNA were detected by a Nano-Drop ND 2000c spectrophotometer (ThermoFisher Scientific, Waltham, MA, USA). The integrity of RNA was measured using both spectrophotometry and agarose gel electrophoresis. 1.0 µg RNA was used to synthesis complementary DNA in a 20 µL reverse-transcription reaction using PrimeScriptTM RT reagent kit (TaKaRa, Dalian, China) with a gDNA eraser. All the primers (Table 1) including target genes and housekeeping gene (*β-actin*) were picked using the NCBI online design instrument based on the certain exon-exon boundaries of published pig gene sequences, and synthesized in Invitrogen Biotechnology company Limited (Shanghai, China). Real-time PCR was measured by a CFX96TM Real-time PCR detection system (Bio-Rad, Hercules, CA, USA) using a SYBR Green PCR reagent kit (TaKaRa, Dalian, China). The reaction system (20 µL) contained 10 µL SYBR Premix Ex Taq II, 2 µL cDNA template, 1 µL forward primer, 1 µL reverse primer, 6 µL ultrapure water. The PCR procedure included pre-denaturation at 95 °C for 30 s, 40 cycles at 95 °C for 5 s and optimal annealing temperature for 30 s, one cycle at 95 °C for 15 s, and the melting curve followed an increasing temperature from 65 °C to 95 °C at a rate of 0.5 °C/s. The relative expression levels of mRNA were calculated using the 2^−ΔΔCT^ method.

### 2.6. Statistical Analysis

All data analyses were performed using SPSS 17.0 (IBM, Armonk, NY, USA). All data were analyzed by a paired Student’s *t*-test comparison between LBW and NBW piglets (*n* = 10), and results are presented as means ± standard errors (SEM). *p* < 0.05 was considered to be a significant difference and values between 0.05 and 0.10 to indicate a trend.

## 3. Results

### 3.1. Birth Weight and Small Intestine Index

Birth weight and small intestine index are shown in Table 2. LBW significantly decreased the birth weight, intestinal weight and length compared with NBW neonates (*p* < 0.05). The relative length of the small intestine was higher in NBW piglets than LBW neonates (*p* < 0.05). The intestinal weight/intestinal length was significantly decreased in LBW piglets.

### 3.2. Small Intestine Morphology

Small intestine morphology data are summarized in Table 3. LBW had a trend to decrease the jejunal villus height (23.3% lower, *p* = 0.09) also the jejunal villi height too (21.2% lower, *p* = 0.09) compared with NBW piglets. LBW significantly decreased villus height/crypt depth of the jejunum compared with NBW piglets (*p* < 0.05).

### 3.3. Gene Expression of Tight Junction Protein in Jejunum

**Figure 1:**
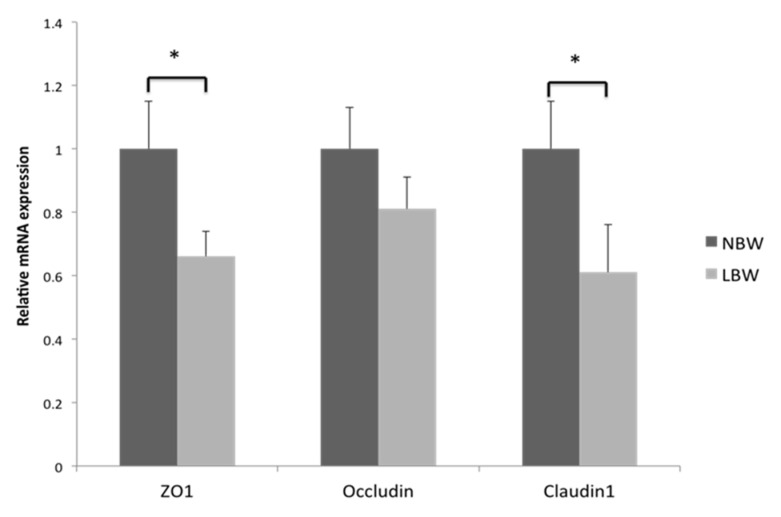
Effects of LBW on mRNA expression of claudin 1, occludin and ZO1 in the jejunum. Data are means ± SEM (*n* = 10 for each group). the bars (*) indicate statistical significance (*p* < 0.05) of gene expression between the two groups. NBW, normal birth weight; LBW, low birth weight; ZO1, zonula occluden-1.

To further investigate the influence of LBW on the physical barrier of the jejunum, we measured the gene expression of tight junction proteins in the jejunum (Figure 1). Compared with NBW piglets, LBW significantly decreased the expression of ZO1 and claudin1 in jejunum (*p* < 0.05).

### 3.4. Redox Genes Expression in Jejunum

The results of redox genes expression in the jejunum are shown in Table 4. Compared with NBW newborn piglets, LBW significantly decreased mRNA expression of *SOD*, *GPX*, *CAT* and *HO-1* (*p* < 0.05) in jejunum.

### 3.5. Histomorphology of Mitochondria

**Figure 2:**
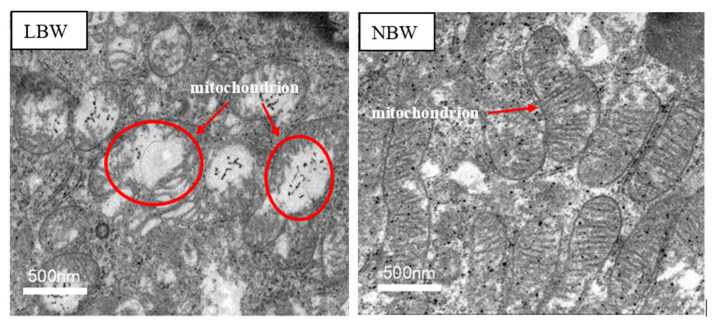
Transmission electron micrographs of the effects of LBW on the histomorphology of jejunal mitochondria. NBW, normal birth weight. LBW, low birth weight. Width of photomicrograph field is 500 nm.

Figure 2 shows the histomorphology of jejunal mitochondria in LBW and NBW newborn piglets. The transmission electron microscope showed that some mitochondria of the epithelial cells of the jejunum demonstrated reduction of the mitochondrial crest and matrix density or even vacuolation and swelling in LBW newborn piglets.

### 3.6. Antioxidant Capacity in Jejunal Mitochondria

The data of antioxidant capacity in jejunal mitochondria is shown in Table 5. LBW significantly decreased the activity of SOD and CAT in jejunal mitochondria relative to the NBW group (*p* < 0.05). LBW tended to increase the content of MDA in jejunal mitochondria compared with the NBW group (60.9% higher, *p* = 0.06).

### 3.7. mRNA Levels of Mitochondria Biogenesis and Function-Related Gene in Jejunum

mRNA levels of mitochondria biogenesis and function-related genes in the jejunum of piglets are presented in Table 6. Expression levels of genes encoding for *PGC-1α* (*p* < 0.05), *Cytc* (*p* < 0.1) and *Ccox IV* (*p* < 0.1) were decreased in LBW piglets compared with NBW piglets. However, there was no significance in jejunal mRNA abundance of *TFAM*, *Nrf1*, *Ccox I* and *ATPS* between LBW newborn piglets and NBW newborn piglets.

## 4. Discussion

LBW has a high incidence in pig production, and previous studies have shown that LBW can cause intestinal dysfunction and oxidative stress in piglets [1,17,18]. In the present study, we found that the intestinal morphology and barrier function of LBW piglets were impaired, and the antioxidant capacity decreased. Furthermore, the present study showed that the mitochondria of the jejunum in LBW piglets were subjected to oxidative stress, and the disturbed mitochondrial morphology and structure may be the main cause of oxidative stress in the jejunum of LBW piglets.

The intestinal tract is an important organ responsible for digestion, absorption and metabolism of dietary nutrients. It contributes 9–12% of the whole body protein synthesis [19] and is the most important route of entry for foreign antigens, including food proteins, commensal gut flora, and invading pathogens [20]. The maintenance of intestinal function is associated with the normal intestinal morphology and structure, healthy intestinal barrier integrity and redox status. However, the present study showed that the small intestine weight/length and the jejunal villus height/crypt depth of LBW newborn piglets were significantly lower than that of NBW newborns, which was in accordance with previous studies [8,21]. It revealed that the small intestine of LBW piglets was longer and thinner, indicating that LBW damaged the intestinal morphology of piglets and affected the growth and development of the small intestine. In addition, the intestinal epithelial barrier plays an important role in maintaining intestinal health and is the first line of defense against a hostile environment within the intestinal lumen [22]. The intestinal barrier is mainly formed by a layer of epithelial cells joined together by tight junction proteins [23], including ZOs, claudins and occludin, which play a central role in the composition and function of tight junctions. They are important protein molecules that constitute the intestinal mucosal barrier and determine intestinal permeability. As the crucial component protein of tight junction, the expressions of *ZO 1*, *occludin* and *claudin-1* are important indicators and methods of indirectly investigating the changes of intestinal permeability [24]. In our study, we observed a decrease of *ZO 1* and *claudin-1* protein expression in the jejunum of LBW piglets compared with NBW newborn piglets. In line with this, Wang et al. found that neonates with intrauterine growth restriction (IUGR) had higher tight-junction permeability and significantly reduced occludin expression [11], suggesting that the intestinal barrier was impaired in LBW piglets.

On the other hand, newborns are vulnerable to oxidative stress due to an underdeveloped antioxidant system [13,25]. Hence, we hypothesized that the abnormal development of the small intestine in LBW neonates is associated with the change of redox status. The results of redox-sensitive gene expression in the jejunum demonstrated that when compared with NBW newborn piglets, LBW subjects had lower mRNA expression of *SOD*, *GPX*, *CAT* and *HO-1*. Among them, *SOD*, *GPX*, *CAT* are important antioxidant enzymes, which can eliminate ROS from the body, thus avoiding oxidative stress [26,27]. *HO-1* is a ubiquitous stress protein, which is able to degrade heme into bilirubin, carbon monoxide and iron [28]. It has been shown that *HO-1* plays an important role in alleviating oxidative stress in intestinal epithelial cells [29]. In line with this study, Wang et al. found that the activities of SOD, GPX and CAT in the jejunum of LBW piglets were significantly lower than that in NBW piglets [30], indicating that LBW piglets suffered oxidative stress, and reduced mRNA expression of *HO-1* gave further proof for altered antioxidant protection in the case of LBW. Taken together, we can assume that oxidative stress in the small intestine of LBW piglets may be one of the vital causes of intestinal dysfunction.

Because mitochondria are closely associated with antioxidant protection [31], we observed the morphological structure of mitochondria in LBW and NBW piglets, and determined the expression of genes related to mitochondrial biosynthesis and function. In the present study, reduced mitochondrial crest and matrix density were observed by transmission electron microscopy in LBW piglets, and some of them even appeared to show vacuolation and swelling, suggesting the morphology and structure of jejunal mitochondria in LBW piglets were impaired. A recent study found that the mitochondria in the intestinal mucosa of postnatal growth retardation piglets aged 42 days was swollen, and the body weight of piglets was significantly lower than that of normal piglets [32]; however, whether it is related to LBW remains to be elucidated. Moreover, we saw that LBW significantly decreased the activity of SOD in jejunal mitochondria and tended to increase the content of MDA compared with the NBW group. MDA is a product of lipid peroxidation, excessive MDA will disturb the redox environment of the intestine, subsequently impairing the structure and function of the small intestine [33]. Hence, the observed increase of MDA and reduced activity of SOD indicated LBW resulted in destruction of the antioxidant system. Accordingly, we determined the expression of *PGC-1α* and important enzymes in respiratory chain. As a vital intracellular signal molecule, *PGC-1α* plays an important protective role against oxidative stress-related diseases [34] and can regulate mitochondrial function by interacting with downstream genes such as *Nrf1* and *TFAM* [35,36]. In addition, *Cytc*, *Cox V*, *Cox IV* and *ATPS* are significant enzymes in the respiratory chain transmission. The down regulation of these enzymes showed a decrease of electron transport function in mitochondria [37,38]. In our study, expression levels of genes encoding for *PGC-1α*, *Cytc* and *Ccox IV* were decreased in LBW piglets compared to NBW piglets. Similarly, a previous study [39] has shown that compared with NBW piglets, IUGR down regulated the mRNA gene abundance of *PGC-1α*, *Ccox V*, and *Cytc* in the jejunum of piglets, and the gene abundance of *NRF1*, *TFAM*, *ATPS* were down regulated too, which was inconsistent with the present study. This may be partly because of the maternal difference and the slaughtering age of piglets. Therefore, further studies need to be done to elucidate these differences. Collectively, these results suggested that oxidative stress in the intestine of LBW piglets may be partly associated with the impaired morphology and functions of mitochondria in the jejunum.

## 5. Conclusions

In summary, the results of the present study showed that LBW piglets exhibited abnormal development of the small intestine, impaired intestinal epithelial barrier functions and disturbed redox status when compared with NBW piglets. However, the impaired mitochondrial morphology and functions may be the cause of intestinal dysfunction in LBW piglets. Taken together, these results provided insights for the mechanisms of intestinal dysfunction in LBW piglets. Overall, the present study laid the foundation for the further study of finding potential solutions to alleviate the adverse effects of LBW.

## Figures and Tables

**Table 1:** Primer sequences used for the real-time polymerase chain reaction (PCR) analysis.

Genes ^1^	Primers	Sequence (5′-3′)	Size (bp)	Accession No.
*SOD*	Forward	GAGACCTGGGCAATGTGACT	139	E06791.1
	Reverse	CTGCCCAAGTCATCTGGTTT		
*CAT*	Forward	TGTACCCGCTATTCTGGGGA	119	NM_214301.2
	Reverse	TCACACAGGCGTTTCCTCTC		
*GPX*	Forward	GCTCGGTGTATGCCTTCTCT	103	AF532927.1
	Reverse	AGCGACGCTACGTTCTCAAT		
*HO-1*	Forward	GCTGAGAATGCCGAGTTCAT	142	NM_001004027.1
	Reverse	TGTAGACCGGGTTCTCCTTG		
*PGC-1α*	Forward	CCCGAAACAGTAGCAGAGACAAG	111	NM_213963
	Reverse	CTGGGGTCAGAGGAAGAGATAAAG		
*TFAM*	Forward	GGTCCATCACAGGTAAAGCTGAA	167	NM_001130211.1
	Reverse	ATAAGATCGTTTCGCCCAACTTC		
*Nrf1*	Forward	GCCAGTGAGATGAAGAGAAACG	166	AK237171.1
	Reverse	CTACAGCAGGGACCAAAGTTCAC		
*NQO1*	Forward	CATACTCCAATGAAGACTATGACAGG	133	NC_010448.4
	Reverse	AAGTTCCAGCTTTTCTACACGC		
*Occludin*	Forward	CAGGTGCACCCTCCAGATTG	110	NM_001162647.2
	Reverse	GGACTTTCAAGAGGCCTGGAT		
*Claudin1*	Forward	GCCACAGCAAGGTATGGTAAC	140	FJ873109.1
	Reverse	AGTAGGGCACCTCCCAGAAG		
*ZO1*	Forward	CTGAGGGAATTGGGCAGGA	105	XM_005659811.1
	Reverse	TCACCAAAGGACTCAGCAGG		
*Cytc*	Forward	AGTTGGCCACCGCCTTATTT	126	NM_001129970
	Reverse	CCAACAGAAACATTCCATCAGCC		
*Ccox I*	Forward	ATTATCCTGACGCATACACAGCA	127	AJ950517.1
	Reverse	GCAGATACTTCTCGTTTTGATGC		
*Ccox IV*	Forward	CCAAGTGGGACTACGACAAGAAC	160	AY786556.1
	Reverse	CCTGCTCGTTTATTAGCACTGG		
*Ccox V*	Forward	GGACCTCATAAGGAAATCTACCCC	123	NC_010449.5
	Reverse	ACACTTTGTCAAGGCCCAGT		
*ATPS*	Forward	TGTCCTCCTCCCTATCACCATT	116	AK230503
	Reverse	TAGTGGTTATGACGTTGGCTTGA		
*Nrf2*	Forward	GAAAGCCCAGTCTTCATTGC	190	XM_003133500.5
	Reverse	TTGGAACCGTGCTAGTCTCA		
*Hsp70*	Forward	GCCCTGAATCCGCAGAATA	152	NC_010446.5
	Reverse	TCCCCACGGTAGGAAACG		
*β-actin*	Forward	TCTGGCACCACACCTTCT	114	DQ178122
	Reverse	TGATCTGGGTCATCTTCTCAC		

^1^*SOD*, superoxide dismutase; *CAT*, catalase; *GPX*, glutathione peroxidase; *HO-1*, heme oxygenase-1; *PGC-1α*, PPARγ coac tivator-1α; *TFAM*, transcriptional factor A; *Nrf1*, nuclear respiratory factor; *ZO1*, zonula occluden-1; *Cytc*, cytochrome c; *Ccox I*, cytochrome c oxidase l; *Ccox IV*, cytochrome c oxidase IV; *ATPS*, Adenosine triphosphate synthase; *Nrf2*, nuclear factor-E2-related factor2; *Hsp70*, heat shock protein 70.

**Table 2:** Effects of LBW on the birth weight and small intestine index in newborn piglets ^1^.

Item	NBW ^2^	LBW ^3^	*p*-Value
Birth weight (kg)	1.66 ± 0.07	0.83 ± 0.04	<0.01
Intestinal weight (g)	43.83 ± 3.07	21.74 ± 1.61	<0.01
Intestinal length (cm)	343.8 ± 5.89	261.8 ± 7.23	<0.01
Intestinal weight/length (g/cm)	0.13 ± 0.01	0.08 ± 0.01	<0.01

^1^ Values are means ± SEM of 10 piglets per group; SEM, standard error of the mean. ^2^ NBW, normal birth weight. ^3^ LBW, low birth weight.

**Table 3:** Effects of LBW on the small intestine morphology in newborn piglets ^1^.

Item	NBW ^2^	LBW ^3^	*p*-Value
Duodenum			
Villus height (µm)	343.44 ± 31.68	311.62 ± 14.73	0.32
Crypt depth (µm)	86.08 ± 14.02	88.43 ± 11.66	0.86
V/C ^4^	4.68 ± 0.54	3.87 ± 0.30	0.16
Jejunum			
Villus height (µm)	428.68 ± 26.84	328.90 ± 33.20	0.09
Crypt depth (µm)	69.11 ± 5.65	76.33 ± 9.30	0.59
V/C	6.50 ± 0.45	4.50 ± 0.33	0.01
Ileum			
Villus height (µm)	386.37 ± 31.14	304.34 ± 26.10	0.09
Crypt depth (µm)	83.97 ± 9.44	82.46 ± 11.40	0.92
V/C	5.00 ± 0.37	4.10 ± 0.41	0.17

^1^ Values are means ± SEM of 10 piglets per group; SEM, standard error of the mean. ^2^ NBW, normal birth weight. ^3^ LBW, low birth weight. ^4^ V/C, villus height/crypt depth.

**Table 4:** Effects of LBW on the expression of redox genes in jejunum of newborn piglets ^1^.

Item ^2^	NBW ^3^	LBW ^4^	*p*-Value
*SOD*	1.00 ± 0.13	0.53 ± 0.13	0.04
*GPX*	1.00 ± 0.15	0.55 ± 0.08	0.05
*CAT*	1.00 ± 0.13	0.57 ± 0.10	0.01
*HO-1*	1.00 ± 0.08	0.79 ± 0.07	0.03
*NQO1*	1.00 ± 0.14	0.71 ± 0.12	0.22
*Nrf2*	1.00 ± 0.08	0.76 ± 0.16	0.31
*Hsp70*	1.00 ± 0.28	1.01 ± 0.31	0.98

^1^ Values are means ± SEM of 10 piglets per group; SEM, standard error of the mean. ^2^ *SOD*, superoxide dismutase; *GPX*; glutathione peroxidase; *CAT*, catalase; *HO-1*, heme oxygenase 1; *NQO1*, NADPH quinine oxidoreductase1; *Nrf2*, nuclear factor-E2-related factor2; *Hsp70*, heat shock protein 70. ^3^ NBW, normal birth weight. ^4^ LBW, low birth weight.

**Table 5:** Effects of LBW on the redox in jejunal mitochondria of newborn piglets ^1^.

Item ^2^	NBW ^3^	LBW ^4^	*p*-Value
MDA (nmol/mg prot)	2.20 ± 0.30	3.54 ± 0.67	0.06
SOD (U/mg prot)	48.42 ± 2.76	41.65 ± 1.24	0.03
CAT (U/mg prot)	7.78 ± 1.00	10.07 ± 1.19	0.10

^1^ Values are means ± SEM of 10 piglets per group; SEM, standard error of the mean. ^2^ MDA, malondialdehyde; SOD, superoxide dismutase; CAT, catalase; nmol/mg prot, nmol/mg protein. ^3^ NBW, normal birth weight. ^4^ LBW, low birth weight.

**Table 6:** Effects of LBW on the mRNA expression levels of mitochondrial-related genes ^1^.

Item ^2^	NBW ^3^	LBW ^4^	*p*-Value
*PGC-1α*	1.00 ± 0.09	0.69 ± 0.06	0.03
*TFAM*	1.00 ± 0.11	0.83 ± 0.08	0.17
*Nrf1*	1.00 ± 0.18	1.00 ± 0.21	1.00
*Cytc*	1.00 ± 0.10	0.65 ± 0.09	0.08
*Ccox I*	1.00 ± 0.11	0.90 ± 0.10	0.61
*Ccox IV*	1.00 ± 0.15	0.57 ± 0.09	0.07
*Ccox V*	1.00 ± 0.17	0.56 ± 0.09	0.10
*ATPS*	1.00 ± 0.16	1.16 ± 0.20	0.48

^1^ Values are means ± SEM of 10 piglets per group; SEM, standard error of the mean. ^2^ *PGC-1α*, PPARγ coactivator-1α; *TFAM*, mitochondria transcriptional factor A; Nrf1, nuclear respiratory factor 1; *Cytc*, cy tochrome c; *Ccox I*, cytochrome c oxidase I; *Ccox IV*, cytochrome c oxidase IV; *Ccox V*, cytochrome c oxidase V; *ATPS*, Adenosine triphosphate synthase. ^3^ NBW, normal birth weight. ^4^ LBW, low birth weight.

## Data Availability

The datasets used to support the findings of this study are available from the corresponding author upon request.
